# The *Polg* Mutator Phenotype Does Not Cause Dopaminergic Neurodegeneration in *DJ-1*-Deficient Mice^1^,^2^,^3^

**DOI:** 10.1523/ENEURO.0075-14.2015

**Published:** 2015-03-23

**Authors:** David N. Hauser, Christopher T. Primiani, Rebekah G. Langston, Ravindran Kumaran, Mark R. Cookson

**Affiliations:** Cell Biology and Gene Expression Section, Laboratory of Neurogenetics, National Institute on Aging, National Institutes of Health, Bethesda, Maryland 20892

**Keywords:** DJ-1, mtDNA, neurodegeration, parkinsonism, Polg mutator, substantia nigra

## Abstract

Mutations in the *DJ-1* gene cause autosomal recessive parkinsonism in humans. Several mouse models of *DJ-1* deficiency have been developed, but they do not have dopaminergic neuron cell death in the substantia nigra pars compacta (SNpc). Mitochondrial DNA (mtDNA) damage occurs frequently in the aged human SNpc but not in the mouse SNpc. We hypothesized that the reason *DJ-1*-deficient mice do not have dopaminergic cell death is due to an absence of mtDNA damage. We tested this hypothesis by crossing *DJ-1*-deficient mice with mice that have similar amounts of mtDNA damage in their SNpc as aged humans (*Polg* mutator mice). At 1 year of age, we counted the amount of SNpc dopaminergic neurons in the mouse brains using both colorimetric and fluorescent staining followed by unbiased stereology. No evidence of dopaminergic cell death was observed in *DJ-1*-deficient mice with the *Polg* mutator mutation. Furthermore, we did not observe any difference in dopaminergic terminal immunostaining in the striatum of these mice. Finally, we did not observe any changes in the amount of GFAP-positive astrocytes in the SNpc of these mice, indicative of a lack of astrogliosis. Altogether, our findings demonstrate the *DJ-1*-deficient mice, *Polg* mutator mice, and *DJ-1*-deficient *Polg* mutator mice have intact nigrastriatal pathways. Thus, the lack of mtDNA damage in the mouse SNpc does not underlie the absence of dopaminergic cell death in *DJ-1*-deficient mice.

## Abstract (precis)

Parkinson’s disease research has been hampered by the absence of animal models that replicate the disease phenotypes observed in humans. We hypothesized that the reason mice lacking *DJ-1*, a gene that causes parkinsonism when mutated, do not replicate the human phenotype is because mice do not have the same levels of mtDNA damage that humans do.

## Significance Statement

Parkinson’s disease research has been hampered by the absence of animal models that replicate the disease phenotypes observed in humans. We hypothesized that the reason mice lacking *DJ-1*, a gene that causes parkinsonism when mutated, do not replicate the human phenotype is because mice do not have the same levels of mtDNA damage that humans do. We tested this hypothesis by crossing *DJ-1*-deficient mice with mice that develop similar amounts of mtDNA damage as humans. We found that the added stress of mtDNA damage does not cause the *DJ-1***-**deficient mice to replicate the human phenotype. These data should be informative for the development of future animal models of Parkinson’s disease.

## Introduction

Early onset autosomal recessive parkinsonism is caused by mutations in the *parkin, PINK1*, and *DJ-1* genes (Kitada et al., 1998; Bonifati et al., 2003; Valente et al., 2004). *PINK1* and *parkin* have been shown to maintain mitochondrial quality control (Corti and Brice, 2013). While the precise biological function of *DJ-1* is unknown, it is known to respond to oxidative stress and defend against mitochondrial damage (Wilson, 2011). Since mitochondrial dysfunction and oxidative stress are features of Parkinson’s disease (PD) (Hauser and Hastings, 2013), studying the functions of *PINK1*, *parkin*, and *DJ-1* may lead to insights about the pathogenesis of sporadic Parkinson’s disease.

In the last decade, several independent lines of *DJ-1* knockout mice have been generated and characterized by multiple groups (Chen et al., 2005; Goldberg et al., 2005; Kim et al., 2005; Manning-Boğ et al., 2007; Chandran et al., 2008; Pham et al., 2010; Rousseaux et al., 2012). In most cases, there was no evidence of dopaminergic cell death in the substantia nigra, with the exception of one of the more recent studies that observed it in a subset of *DJ-1^−/−^* mice (Rousseaux et al., 2012). The general lack of dopamine neuronal degeneration has also been reported for *parkin* knockout (Goldberg et al., 2003; Itier et al., 2003), *PINK-1* knockout (Kitada et al., 2007), and triple *DJ-1*/*parkin*/*PINK-1* knockout mice (Kitada et al., 2009).

The reasons underlying the phenotypic discrepancies between mouse models of autosomal recessive parkinsonism and the humans that have these diseases are not known. It is possible that mouse substantia nigra pars compacta (SNpc) neurons deficient for the autosomal recessive PD genes do not degenerate because they are not exposed to the same types of stressors that human SNpc neurons are. One such stressor is mitochondrial DNA (mtDNA) damage, which accumulates with age at high levels in the human SNpc (Bender et al., 2006; Kraytsberg et al., 2006). *Polg* mutator mice develop mtDNA damage as they age due to a knock-in proofreading-deficient version of the mtDNA polymerase gamma (Kujoth et al., 2005). By the time they reach 1 year of age, ∼50% of mtDNA molecules found in SNpc neurons of the *Polg* mutator mice have deletions, which is comparable to that observed in the aged human SNpc (Bender et al., 2006; Kraytsberg et al., 2006; Perier et al., 2013). This increased mtDNA damage results in a decrease in the abundance of mitochondrial respiratory chain complex I subunits in *Polg* mutator brains (Hauser et al., 2014). Since *DJ-1* protects against complex I inhibition both *in vitro* (Mullett and Hinkle, 2011) and *in vivo* (Kim et al., 2005), we hypothesized that increasing mtDNA damage in the *DJ-1* knockout mouse SNpc would result in neurodegeneration. We tested this hypothesis by crossing *DJ-1* knockout mice with *Polg* mutator mice.

## Materials and Methods

### *DJ-1;Polg* mice

This study was carried out in strict accordance with the recommendations in the Guide for the Care and Use of Laboratory Animals of the National Institutes of Health. The protocol was approved by the Institutional Animal Care and Use Committees of the US National Institute of Child Health and Human Development (Animal study protocol number 12-059).

The *Polg* mutator mice used in this study were originally described by Kujoth and colleagues (2005). *DJ-1* knockout mice were generated and originally characterized by Chandran and colleagues (2008) and given to us after having been backcrossed at least two generations into C57BL/6J. We backcrossed the *DJ-1* mice for an additional three generations into C57BL/6J prior to mating one *DJ-1^+/−^* mouse with one *Polg^WT/MT^* mouse. *DJ-1^+/−^;Polg^WT/MT^* mice were then bred with each other to produce the cohorts of mice used in this study. All of the mice were given access to food and water *ad libitum*.

Four genotypes (*DJ-1^+/+^;Polg^WT/WT^*, *DJ-1^+/+^;Polg^MT/MT^*, *DJ-1^−/−^;Polg^WT/WT^*, and *DJ-1^−/−^;Polg^MT/MT^*) of mice were aged to at least 1 year (365-391 d, median 377 d) before transcardial perfusion. One male *DJ-1^+/+^;Polg^MT/MT^* mouse and one male *DJ-1^−/−^;Polg^MT/MT^* mouse were euthanized at the ages of 340 and 352 d, respectively, at the request of the veterinarians due to the severity of their phenotype. One female *DJ-1^+/+^;Polg^MT/MT^* mouse was euthanized at 365 d at the request of the veterinarians due to an ear infection. These mice were not used for weight analysis or the pole test but were used for immunohistochemistry.

### Pole Test

We performed the pole test as previously described (Ogawa et al., 1985; Matsuura et al., 1997). A wooden dowel (1 cm diameter, 0.5 m height) was mounted into a wooden base and the entire apparatus was placed into an empty mouse cage and covered with fresh bedding. The mice were placed at the top of the pole and video recorded as they descended. Several pretrials were done before a series of four to seven trials were recorded for each animal. Some animals were given an intraperitoneal injection of l-DOPA (25 mg/kg) and benserazide (5 mg/kg) after their first set of trials and then subjected to four to seven more trials 30 min following the injection. After all the mice had been tested, an operator that was blinded to both the genotype and drug treatment of the mice scored the video files. The operator recorded the time it took the mice to reach the floor of the cage after being placed atop the pole along with their method of doing so (Walk, Slide, Walk/Slide, or Fall). A mouse was judged to have fallen if it fell to the cage floor at any point of its descent.

### Immunohistochemistry

Mice were transcardially perfused using PBS (1 min) and then 4% PFA in PBS (5 min). After perfusion, brains were removed and postfixed overnight in 4% PFA in PBS at 4 °C. The brains were then transferred into a solution of 30% w/v sucrose in PBS that was supplemented with 0.05% sodium azide and stored at 4 °C until the brains had sunk to the bottom of the containers. Each brain was then bisected along the longitudinal fissure and the left hemisphere was sectioned on a cryostat into 40-μm-thick sections. Slices that included the midbrain were collected and stored individually, while sections rostral and caudal to the midbrain were stored in groups. For stereology, every fourth section through the midbrain was stained for glial fibrillary acidic protein (GFAP) and/or tyrosine hydroxylase (TH) immunoreactivity using a free-floating procedure in which all steps were performed on a rotating shaker (∼250 rpm).

For the 3,3′-diaminobenzadine (DAB) staining protocol, the sections were incubated in 0.3% hydrogen peroxide in PBS for 20 min at room temperature (RT) then washed three times with PBS. Sections were blocked for 1 h at RT in blocking buffer [PBS supplemented with 1% w/v bovine serum albumin (BSA), 0.3% Triton X-100, and 1% donkey serum], which was also used to dilute primary and secondary antibodies in subsequent steps. Sections were then incubated overnight in primary TH antibody (PelFreez # P40101, rabbit polyclonal, 1:2000 dilution) at 4 °C. The next day, sections were left in primary antibody for 1 h at RT then washed with PBS three times for 5 min. A biotinylated secondary antibody (Vector Labs #BA1100, horse anti-rabbit IgG, 1:500 dilution) was incubated with the sections for 1 h at RT then the slices were washed three times for 5 min with PBS. The sections were then exposed to 0.3% hydrogen peroxide in PBS for 20 min then washed again with PBS three times for 5 min each. The slices were then incubated with a mixture of avidin and biotinylated horseradish peroxidase (Vector Labs Vectastain Universal Elite ABC kit, product #PK-6200) for 20 min at RT then washed three times with PBS for 10 min per wash. To complete the staining procedure, the slices were incubated with a DAB peroxidase substrate (Vector Labs # SK-4100) for 5 min and washed with PBS. Finally, the sections were mounted onto slides and dehydrated with a series of ethanol washes followed by two washes in xylenes, and then coverslips were added using Eukitt mounting media.

For the fluorescent staining protocol, the sections were washed with 1× PBS three times each for 10 min at room temperature on shaker. Sections were blocked for 1 h at RT in blocking buffer (PBS supplemented with 1% w/v BSA, 0.3% Triton X-100, and 1% v/v donkey serum), which was used to dilute primary and secondary antibodies in later steps. Sections were then incubated overnight in primary TH antibody (PelFreez # P40101, rabbit polyclonal, 1:2000 dilution) and GFAP antibody (BD Pharmingen #556329, mouse monoclonal, 1:1000 dilution) at 4 °C. The following day, sections were rinsed in 1× PBS three times for 10 min each. Two secondary antibodies were incubated with the sections (Alexa Fluor #A21206, 488 donkey anti-rabbit IgG, 1:500 dilution; and Alexa Fluor #A10037, 568 donkey anti-mouse IgG, 1:500 dilution) for 2 h at RT and protected from light. The slices were then washed three times for 10 min each in 1× PBS before being mounted on glass slides using Prolong Gold mounting media.

Stereology was performed on a Zeiss Axio Imager A1 microscope running Stereo Investigator software (MBF Biosciences). An operator blinded to the genotype of each sample operated the microscope and performed stereology. Unbiased counting of the SNpc TH- and GFAP-positive cells was accomplished using the software’s optical fractionator protocol. As only the SNpc of the left hemisphere was analyzed, the cell counts were multiplied by two to estimate whole-brain SNpc cell numbers.

In order to determine striatal TH terminal density, three sections through the striatum were stained per animal, with one *DJ-1^+/+^;Polg^MT/MT^* animal removed from this analysis because its striatum was sectioned at a different thickness than all other animals. The sections were stained for TH as described above, except in this case a different secondary antibody was used (Jackson ImmunoResearch #711-655-152, Alexa-Fluor 790 AffiniPure Donkey anti-rabbit IgG, 1:1000).

To quantitatively image the sections, all of the slides were scanned at once using an Odyssey CLx imaging system. The highest resolution (21 μm) and scan quality settings were used, and the system’s automatic intensity feature was employed to avoid pixel saturation. The signal intensity was measured inside an equally sized circle placed approximately in the same area of the dorsal striatum of each slice. The mean intensity of the sections from each animal was used for comparisons.

### Statistics

*Post hoc* power analysis was done using the “pwr” package in R (http://www.R-project.org/). Sample sizes were the minimum group size, the effect size was 0.25 (Cohen, 1988), and the *p* values calculated from the ANOVA or χ^2^ tests were used to determine *post hoc* power values. These values are reported in Table 1.

**Table 1:** Statistical table

	Data structure	Type of test	Power (*f* = 0.25)
a		Χ^2^	0.999
b	*N* too small to determine if normally distributed	One-way ANOVA	0.0001
c	*N* too small to determine if normally distributed	One-way ANOVA	0.471
d	*N* too small to determine if normally distributed for 3 of 4 genotypes. The fourth genotype with 8 animals is normally distributed.	One-way ANOVA	0.274
e	*N* too small to determine if normally distributed for 3 of 4 genotypes. The fourth genotype with 8 animals is normally distributed.	One-way ANOVA	0.632
f	*N* too small to determine if normally distributed for 3 of 4 genotypes. The fourth genotype with 8 animals is normally distributed.	One-way ANOVA	0.493
g	*N* too small to determine if normally distributed for 3 of 4 genotypes. The fourth genotype with 8 animals is normally distributed.	One-way ANOVA	0.350

*Post hoc* power calculations for each statistical test reported are recorded in this table. To calculate *post hoc* power, we used an effect size of 0.25, a sample size that was the minimum group size, and the actual *p* value returned by the indicated test. For almost all instances, we were unable to formally test whether the data were normally distributed because the sample size was not 8 or greater. Superscript letters in the figure legends refer to the first column of the table.

## Results

**Figure 1:**
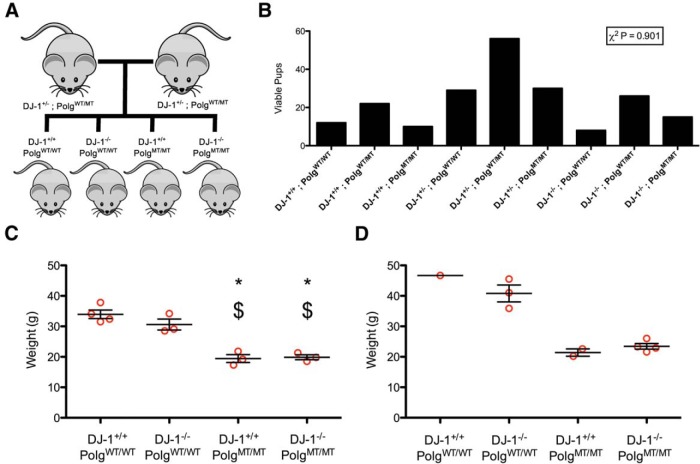
Generation of *DJ-1* knockout *Polg* mutator mice. ***A*,** Double heterozygous mice were bred to generate the four genotypes of mice used in this study. ***B*, **The number of viable pups born from double heterozygous breeding is shown. A χ^2^ test was used to determine that the observed proportions did not differ from the expected proportions (*n* = 208 mice, *p* = 0.901)^a^. ***C*, **The weights of female mice at one year of age are displayed. The groups were compared with ANOVA (*F*_(3,9)_ = 29.17, *p* = 0.00005745)^b^ followed by Tukey’s multiple comparison test (*p < 0.05 vs *DJ-1^+/+^;Polg^WT/WT^*, $*p* < 0.05 vs *DJ-1^−/−^;Polg^WT/WT^*). ***D*, **The weights of male mice at 1 year of age.

We bred double heterozygous *DJ-1^+/−^;Polg^WT/MT^* mice and analyzed the birth rates of the resulting nine genotypes (Fig. 1*A*,*B*). All of the genotypes were born at the anticipated Mendellian ratios (Fig. 1*B*). From the nine possible genotypes of mice, we used the four double homozygous genotypes for subsequent analysis. We aged a cohort of 27 mice (Table 2) for ∼1 year in order to maximize the aging effect of the *Polg* phenotype. We note that this approaches the maximum lifespan of these animals as the *Polg* genotype causes severe weight loss as the animals approach 1 year of age (Kujoth et al., 2005). We weighed our animals after they had reached a year of age to determine if the loss of *DJ-1* had any effect on the weight loss phenotype cause by *Polg* mutation (Fig. 1*C*,*D*). In both males and females, we observed weight loss in the *Polg* mutator animals consistent with previous results (Kujoth et al., 2005). However, in the females, we observed no difference between the *Polg* mutator mice with and without *DJ-1* (Fig. 1*C*). Our cohort did not have enough males to allow for statistical analysis, but the trend of no difference was also apparent in the males (Fig. 1*D*).

**Table 2:** Genotypes and genders of the cohort of mice used for experiments

*DJ-1*	*Polg*	Male	Female	Total
+/+	WT/WT	1	5	6
−/−	WT/WT	3	4	7
+/+	MT/MT	3	3	6
−/−	MT/MT	5	3	8

The genotype and gender of the cohort of 27 mice used for experiments are recorded in the table.

**Figure 2:**
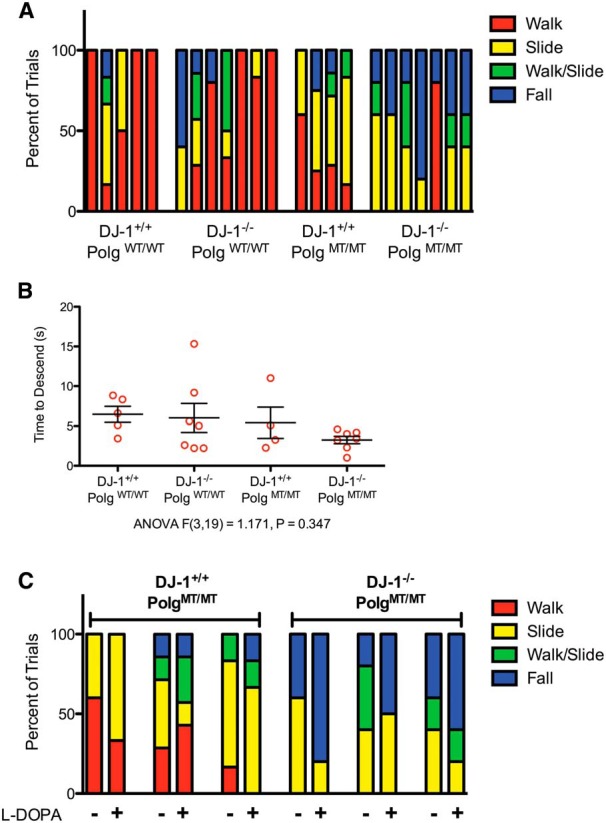
Behavioral characterization using the pole test. ***A***, The mice were tested for behavioral deficits using the pole test and the method of descent for each mouse during each of their trials is displayed. Each bar represents an individual animal, and the methods of descent from four to seven trials are reported as a proportion within the bar. ***B*, **The mean time to descend the pole for each mouse is displayed (*n* = 4-7 mice per genotype, *n* = 4-7 trials per mouse, ANOVA *F*_(3,19)_ = 1.171, *p* = 0.347)^c^. ***C*, **Following their first set of trials on the pole test, three mice in the *DJ-1^+/+^;Polg^MT/MT^* group and three mice in the *DJ-1^−/−^;Polg^MT/MT^* were given l-DOPA and retested 30 min later. The results of the test before and after l -DOPA are displayed with each bar representing an individual animal (*n* = 4-7 trials).

In order to determine if any of the mice had motor impairments that could be indicative of dopamine cell loss, we tested them using the pole test. During this test, the mouse is placed atop a vertical pole and observed as it descends the pole. Mice with SNpc dopamine cell loss caused by 6-hydroxydopamine or 1-methyl-4-phenyl-1,2,3,6-tetrahydropyridine spend significantly longer amounts of time at the top of the pole (Ogawa et al., 1985; Matsuura et al., 1997). Importantly, the behavior of mice lesioned by either drug can be rescued by administration of l-DOPA, which demonstrates that this test is sensitive to dopamine levels (Ogawa et al., 1985; Matsuura et al., 1997). When we tested our mice using this assay, we noticed that mice with the *Polg* mutator mutation tended to slide down or fall off of the pole but did not freeze at the top (Fig. 2*A*). Videos of a *DJ-1^+/+^;Polg^WT/WT^* mouse and a *DJ-1^−/−^;Polg^WT/WT^* mouse performing the task correctly by reorienting themselves and walking down the pole are shown in Movies 1 and 2, respectively. A *DJ-1^+/+^;Polg^MT/MT^* mouse sliding down the pole and a *DJ-1^−/−^;Polg^MT/MT^* falling from the pole can be seen in Movies 3 and 4. When we compared the duration it took the mice to descend, regardless of falls and slides, we found that there was no difference between the genotypes (Fig. 2*B*). This likely reflects the different means by which the animals descended. To determine if dopamine depletion was the underlying reason that the *Polg* mutator genotypes tended to slide and fall, we gave several of them l -DOPA to increase dopamine levels in their brains and tested them again. We found that l -DOPA did not prevent the mice from sliding or falling during the task (Movies 5,6; Fig. 2*C*). Thus, the inability of *Polg* mutator mice, regardless of *DJ-1* genotype, to perform the pole test correctly was likely not due to dopamine deficiency.

Movie 1 Pole test of a *DJ-1^+/+^;Polg^WT/WT^* mouse. The mouse performed the task correctly be reorienting itself and walking down the pole. 10.1523/ENEURO.0075-14.2015.video.1

Movie 2 Pole test of a *DJ-1^−/−^;Polg^WT/WT^* mouse. This mouse performed the task correctly by walking down the pole. 10.1523/ENEURO.0075-14.2015.video.2

Movie 3 Pole test of a *DJ-1^+/+^;Polg^MT/MT^* mouse. The mouse did not perform the task correctly because it did not orient itself downwards and because it slid down the pole. 10.1523/ENEURO.0075-14.2015.video.3

Movie 4 Pole test of a *DJ-1^−/−^;Polg^MT/MT^* mouse. The mouse did not perform the task correctly because it fell from the top of the pole. 10.1523/ENEURO.0075-14.2015.video.4

Movie 5 Pole test of a *DJ-1^+/+^;Polg^MT/MT^* mouse given l-DOPA. This mouse (also shown in Movie 3) was given l -DOPA 30 min prior to the test. It did not perform the task correctly, but instead slid down the pole. 10.1523/ENEURO.0075-14.2015.video.5

Movie 6 Pole test of a *DJ-1^−/−^;Polg^MT/MT^* mouse given l -DOPA. This mouse (also shown in Movie 4) was tested 30 min after l -DOPA administration. It did not perform the task correctly, but instead fell from the top of the pole. 10.1523/ENEURO.0075-14.2015.video.6

**Figure 3:**
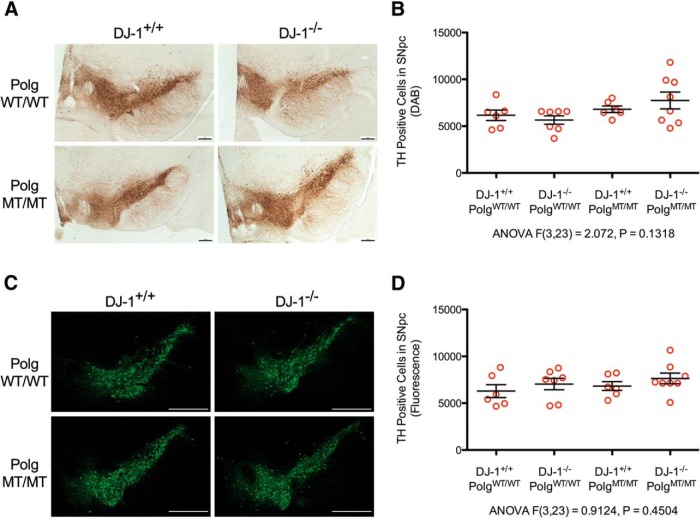
Stereological counts of dopaminergic neurons in the SNpc. Unbiased stereology was performed by a blinded observer to count the number of dopaminergic neurons in the SNpc of the mice after that had reached a year of age. Two separate experiments were performed to analyze the same set of brains. ***A*, **TH immunoreactive cells in the midbrain stained brown using DAB (scale bars, 200 μm). ***B*, **DAB-stained cell counts for each animal (red circles) along with mean and SEM of each group (*n* = 6-8 mice per genotype, ANOVA *F*_(3,23)_ = 2.072, *p* = 0.1318)^d^. ***C*, **TH immunoreactive cells were detected in the midbrain using fluorescence (TH = green; scale bars, 500 μm). ***D*, **The numbers of SNpc dopaminergic neurons counted using stereology for each animal (red circles) are shown with mean and SEM (*n* = 6-8 mice per group, ANOVA *F*_(3,23)_ = 0.9124, *p* = 0.4504)^e^.

After all of the mice had been perfused, we determined the integrity of their nigrastriatal axis using several measures. First, we counted the number of dopaminergic neurons in the SNpc using unbiased stereology. The staining and counting was done blindly, and a prospective power analysis calculated that our study design had a power of 95.5% to detect a 25% change in SNpc TH-positive cells. We performed the stereology experiment twice, once with a colored DAB stain (Fig. 3*A*,*B*) to mark TH-positive neurons and then once with fluorescent detection (Fig. 3*C*,*D*) using a separate group of tissue sections. We reasoned that doing the stereology using two different methods would decrease the likelihood of detecting any false positives. In both experiments, we did not detect any difference in the numbers of SNpc dopaminergic cells between the genotypes of mice.

**Figure 4:**
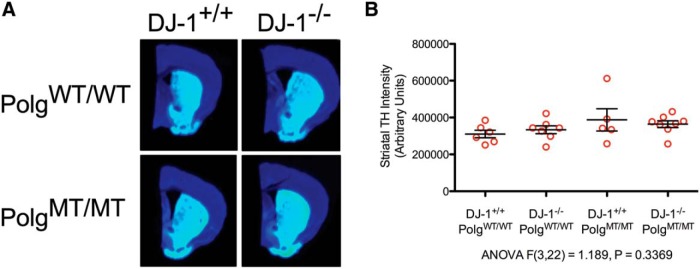
Dopaminergic terminal density in the striatum. ***A***, Representative TH-stained tissue sections through the striatum. The sections were immunostained using an infrared fluorescent dye conjugated secondary antibody and imaged using an infrared imaging system. The sections are pseudo-colored using a heat map, with warmer colors indicating strong TH immunoreactivity. ***B***, Striatal TH staining intensity calculated from infrared imaged tissues. Individual data points represent animals and the mean and SEM are also displayed (three sections per animal were averaged, *n* = 5-8 animals, ANOVA *F*_(3,22)_ = 1.189, *p* = 0.3369)^f^.

In some instances of damage to the nigrastriatal axis, such as methamphetamine toxicity, the cell bodies of SNpc dopaminergic neurons remain alive while their nerve terminals in the striatum degenerate (Ricaurte et al., 1982). To determine if there was any dopaminergic terminal degeneration in any of our mice, we immunostained sections through their striata for TH and quantified the stain intensity using an infrared imaging system. Using this assay, we were unable to detect any changes in striatal TH intensity amongst any of our groups of mice (Fig. 4).

**Figure 5:**
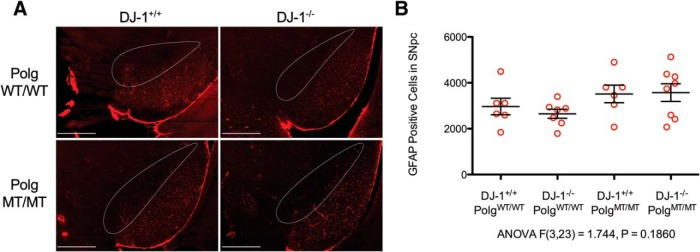
Detection of astrogliosis in the SNpc. ***A*, **GFAP-positive astrocytes were immunostained in the SNpc (outlined in white) and surrounding tissue (GFAP = red; scale bars, 500 μm). Unbiased stereology was used to count GFAP-positive cells in the SNpc simultaneously with the TH cells counts shown in Figure 3*B*. ***B*, **GFAP-positive SNpc cell counts per animal (red circles) along with mean and SEM are displayed in the graph (ANOVA *F*_(3,23)_ = 1.744, *p* = 0.1860)^g^.

Altogether, our data demonstrates that the nigrastriatal axis is intact in aged *Polg* mutator mice with *DJ-1* deficiency. Since *DJ-1* is known to be expressed in astrocytes (Bandopadhyay et al., 2004), we considered whether or not our mice would have phenotypes that manifest themselves in astrocytes. To determine this, we chose to examine the SNpc for astrogliosis as indicated by increased GFAP immunoreactivity. We found no difference when we compared the numbers of GFAP-positive astrocytes in the SNpc between the genotypes (Fig. 5). Therefore, the *Polg* mutator mutation in *DJ-1*-deficient mice does not cause increased astrogliosis in the SNpc.

## Discussion

We hypothesized that crossing *DJ-1*-deficient mice with *Polg* mutator mice in order to increase mtDNA damage in their substantia nigra would result in the degeneration of dopaminergic neurons. Using a cohort of mice designed to test this hypothesis with sufficient statistical power, we were able to demonstrate that our hypothesis was false. We also found that the loss of *DJ-1* had no effect on the weight phenotype of the *Polg* mutator mice, that none of the nine possible combinations of *DJ-1* and *Polg* genotypes were embryonic lethal, and that there was not increased astrogliosis in the *Polg* mutator *DJ-1*-deficient mouse SNpc.

Our results are similar to other studies that have crossed *DJ-1* knockout mice with other knockout mice. The triple knockout of *DJ-1*/*parkin*/*PINK-1* had no effect on SNpc cell numbers in mice up to 24 months of age (Kitada et al., 2009). Similarly, crossing *DJ-1*/*parkin* knockout mice with *GPx1* knockout mice did not result in SNpc degeneration at 18 months of age (Hennis et al., 2014). Likewise, no effect on dopaminergic cell numbers was observed when *DJ-1*/*parkin* knockout mice were crossed with mice deficient for either SOD1 or SOD2 and aged to at least 16 months (Hennis et al., 2013).

Two studies have analyzed the nigrastriatal axis in aged *Polg* mutator mice (Dai et al., 2013; Perier et al., 2013). While both studies found no degeneration of SNpc dopaminergic neuron cell bodies, they reported conflicting results for striatal TH terminal density. One reported a decrease in striatal TH staining in aged *Polg* mutator mice (Dai et al., 2013), while the other did not observe a change in striatal TH (Perier et al., 2013). In our cohort of animals, the *Polg* mutator genotype did not cause SNpc cell loss nor did it cause the loss of striatal TH terminals.

Previous studies have shown that aged *Polg* mutator mice accumulate SNpc mtDNA deletions to a similar extent as that found in the SNpc in both PD patients and aged neurologically normal controls (∼50% of mtDNA molecules harboring deletions) (Bender et al., 2006; Kraytsberg et al., 2006; Perier et al., 2013). In addition, our analysis of the brains of aged *Polg* mutator mice from our own colony demonstrated a loss of respiratory chain proteins, which is indicative of mtDNA damage (Hauser et al., 2014). Since the experiments reported here required the use of fixed tissue, measuring the amount of mtDNA damage in the SNpc of our mice could not be done and is an important future experiment. We note that the *Polg* mutator mice with and without *DJ-1* all developed the premature aging phenotype and the body weights between these two groups were similar (Fig. 1*C*,*D*). This suggests that the absence of *DJ-1* was unlikely to have strongly accelerated mtDNA damage caused by the *Polg* mutation, although we cannot exclude a more subtle effect. Regardless, whatever the level of mtDNA damage that had occurred in these animals, it was not sufficient to induce dopaminergic cell death. Whether or not other genetic manipulations combined with the loss of *DJ-1* lead to SNpc degeneration in mice should be the subject of future studies.

## Synthesis

The decision was a result of the Reviewing Editor Julie Andersen and the peer reviewers coming together and discussing their recommendations until a consensus was reached. A fact-based synthesis statement explaining their decision and outlining what is needed to prepare a revision is listed below. The following reviewer agreed to reveal her identity: Manuela Basso

The two requisite reviews of your manuscript entitled "The Polg mutator phenotype does not cause dopaminergic neurodegeneration in DJ-1 deficient mice" have now been received. I apologize for the delay in review time, but we encountered some difficulty obtaining a sufficient number over the holiday season. I am happy to inform you that the manuscript has been deemed by both reviewers as accepted with only minor revisions required. The manuscript was rated as well-written and a clear demonstration that mitochondrial DNA damage does not act in synergy with DJ-1 repression to elicit dopaminergic neurodegeneration. Although a negative result that does not provide a major advance in the field per se, it does provide important information to other investigators in this research area. Specific comments by the reviewers are listed below:

(1) The authors mention in the Abstract that lack of neurodegeneration in DJ-1 knockout mice is likely due to lack of mtDNA damage. It would be helpful to provide a quantitation of the amount of mtDNA damage in both single and combined mouse models.
(2) There is some concern about how results from the pole test were reported in Figure 2. Watching the video, there is a remarkable difference between Polg WT or KO mice. In the KO, the mice are simply falling down, not descending.
(3) There is a question about the rationale for using both DAB immunohistochemistry and fluorescence for the detection of TH within the SN, could you please clarify?
(4) In the Methods section, due to their similarity TH immunostaining for striatum and SNpc should be combined.

## Author Response

### Response to reviews:

We would like to thank the reviewers for their helpful comments. In response to the numbered suggestions, we have made the following changes to the manuscript:

1. The authors mention in the Abstract that lack of neurodegeneration in DJ-1 knockout mice is likely due to lack of mtDNA damage. It would be helpful to provide a quantitation of the amount of mtDNA damage in both single and combined mouse models.
  Response: We agree with the reviewer that this information would be helpful, and have included new text in the introduction (pg. 4) and final paragraph (pg. 14) that describes the actual percentage of mtDNA deletions others have reported to occur in the Polg mutator mouse SNpc.
  In prior studies from other groups, approximately 50% of mtDNA molecules have deletions as determined using an RT-PCR based assay. Additionally, we have previously detected decreased levels of respiratory chain proteins in the brains of aged Polg mutator mice from our colony, which would indicate mtDNA damage (Hauser et al., 2014). Unfortunately, the RT-PCR method used to estimate mtDNA damage requires fresh tissue, which we did not collect from our cohort of mice. We had decided ahead of time to perfuse and fix tissue for optimal immunostaining so we could evaluate our primary endpoint, TH positive cell loss. Therefore we are unable to determine the degree of mtDNA damage directly in these specific mice at this time. Because we agree that this is important, we have altered the text to include the clarifying statements. First, we state that we have not measured mtDNA damage directly, and that would be an important future experiment. Second, we note that in the experiments reported here, the DJ-1 deficient Polg mutator animals all developed the accelerated aging phenotype and that in the quantitative measure of body weight the DJ-1-/-;PolgMT/MT group was similar to the DJ-1+/+;PolGMT/MT group. This suggests that the absence of DJ-1 was unlikely to have strongly accelerated mtDNA damage caused by the Polg mutation, although we cannot exclude a more subtle effect. Third, whatever the level of mtDNA damage that had occurred in these animals, it was not sufficient to induce dopaminergic cell death, which again was our primary endpoint.
2. There is some concern about how results from the pole test were reported in Figure 2. Watching the video, there is a remarkable difference between Polg WT or KO mice. In the KO, the mice are simply falling down, not descending.
  Response: We completely agree that clarification on what the time to descend tells us is very important. To address this, a blinded observer examined the original videos and recorded the method by which the animal descended. We have added this data to Figure 2 (2A and 2C). We hope that this clarifies that a subset of animals are falling down, although the mean time to descend is not significantly different between groups.
  However, similar to point 1, our view is that the main point of this data is that the mice did not freeze on top the pole, ie show an increase in time taken to descend, as would be expected if there was damage to the nigra. Additionally, L-DOPA did not prevent the mice from falling.
  Therefore, the observations of falling are not related to dopamine depletion and, in our view, are probably related to the diminished bodyweight of the animals carrying the Polg mutation. Again, we really do appreciate the opportunity to present the data and the interpretation in as clear and open manner as we can.
3. There is a question about the rationale for using both DAB immunohistochemistry and fluorescence for the detection of TH within the SN, could you please clarify?
  Response: We originally used the DAB method to determine SNpc cell counts, and once we determined that there were no differences we sought to measure astrogliosis, which required fluorescence detection of both TH and GFAP. We reasoned that repeating the TH cell counts while we counted the GFAP cells would strengthen the findings of the DAB method. In order to alleviate any confusion to the reader, we have added some text to clarify that the experiments were done on the same set of animals (pg 12).
4. In the Methods section, due to their similarity TH immunostaining for striatum and SNpc should be combined.

Response: We agree with the reviewer and have combined the two sections (pg. 6-9).
